# Impact of surgical orthodontic treatment on quality of life in Chinese young adults with class III malocclusion: a longitudinal study

**DOI:** 10.1186/s12903-019-0782-9

**Published:** 2019-06-13

**Authors:** Jiaan Ni, Shaohua Song, Nuo Zhou

**Affiliations:** 1 Orthognathic Centre, College of Stomatology, GuangXi Medical University, Nanning, 530021 People’s Republic of China; 2 Department of Oral and Maxillofacial Surgery, College of Stomatology, GuangXi Medical University, Nanning, 530021 People’s Republic of China

**Keywords:** Class III malocclusion, Orthognathic surgery, Orthodontic treatment

## Abstract

**Background:**

The quality of life in Class III malocclusion patients are worse than those without the disorder and previous studies have shown that surgical orthodontic treatment has a different effect on these patients compared with mild or moderate malocclusion. This study aimed to investigate the changes in quality of life in patients with Class III malocclusion during surgical orthodontic treatment in Chinese young adults.

**Methods:**

The 14-item Short Form Oral Health Impact Profile (OHIP-14), and the 22-item Orthognathic Quality of Life Questionnaire (OQLQ) were used to assess the effect of surgical orthodontic treatment on the quality of life in 21 patients with Class III malocclusion at pre-treatment (*T*_0_), pre-surgical orthodontic treatment (6 to 8 months, *T*_1_) and post-surgical orthodontic treatment (6 to 8 months after surgery, *T*_2_), and 24 healthy individuals were included as controls. The comparisons in numerical variables between patients and controls were performed using Mann-Whitney U test. The scores of the two questionnaires between *T*_0_, *T*_1_, *T*_2_ and controls (*T*c) were compared using generalized estimating equation.

**Results:**

According to OHIP-14 questionnaire, the mean scores in *T*_0_ and *T*_1_ were higher than those in *T*_2_ and *T*c (*P* < 0.001), and a significant decrease was observed after post-surgical orthodontic treatment (*P* < 0.001), which achieved a level similar to the control group (*P* > 0.05). As to OQLQ questionnaire, the mean scores of all domains showed a significant increase between *T*_0_ and *T*_1_ except for awareness of dentofacial aesthetics (*P* > 0.05) and social aspects of dentofacial deformity (*P* > 0.05), followed by a significant decrease between *T*_1_ and *T*_2_.

**Conclusion:**

Surgical orthodontic treatment may improve quality of life in patients with Class III malocclusion, but pre-surgical orthodontic treatment may have an adverse effect on quality of life.

## Background

Class III malocclusion is considered as one of the most complex and intractable orthodontic disorders with a concave profile that exhibits maxillary retrusion, mandibular protrusion or a combination of both. Numerous epidemiological studies have been conducted to illustrate the importance of Class III malocclusion as a complicated disorder, and suggest that the prevalence of Class III malocclusion varies greatly among different populations, of which the Southeast Asian population shows the highest prevalence of 15.8% [1, 2]. According to a cross-sectional study conducted in Chinese school children, the prevalence of Class III malocclusion was relatively higher than other ethnics with a rate of 12.6% [2]. Class III malocclusion significantly affects oral function and facial aesthetics, and there is a tendency for this to worsen with age [3, 4]. Therefore, the high prevalence and adverse effects of Class III malocclusion have made it a serious public health problem.

The concept of quality of life refers to not only the absence of disease, but also the presence of physical, mental and social well-being [5]. However, Class III malocclusion is reported to be one of the most important risk factors for low appearance self-esteem and self-confidence [6]. A systematic review also suggests that quality of life in Class III malocclusion patients is worse than those without this disorder [7]. The improvement of patients’ quality of life is an important indicator for evaluating the outcome of malocclusion treatment [8]. At present, surgical orthodontic treatment is one of the most important methods to treat malocclusion and facial deformities, and the quality of life in patients treated by surgical orthodontic treatment is better than before surgery [9–11]. Advances in technology contribute to more accurate diagnosis, and this means higher diagnostic capabilities to plan treatments for improvements in outcomes.

Surgical orthodontic treatment can correct dentofacial deformities and improve the patients’ social life by improving aesthetics and function [12, 13]. Surgical orthodontic treatment is a well-established treatment for patients with severe dentofacial anomalies. Although many studies have demonstrated that the quality of life in these patients improves after treatments [7, 9–11, 14], limited studies on the change in quality of life during surgical orthodontic treatment have been conducted in the Chinese population, particularly with Class III malocclusion [15–18], and previous studies have shown that surgical orthodontic treatment has a different effect on these patients [17, 19]. The Orthognathic Quality of Life Questionnaire (OQLQ) and the Oral Health Impact Profile (OHIP-14) have been widely used to evaluate the oral health-related quality of life in the world [20, 21]. The OQLQ tool is a brief condition-specific measurement for quality of life [22]. The OHIP-14 is used to evaluate the changes of oral health-related quality of life. This study aimed to determine the changes in quality of life in patients with Class III malocclusion during surgical orthodontic treatment using generic oral health and condition-specific approaches in Chinese young adults.

## Methods

### Study design

A total of 21 patients with Class III malocclusion who had undergone surgical orthodontic treatment were enrolled from the department of orthognathic surgery at the affiliated stomatology hospital of Guangxi Medical University from September 2014 to August 2017. Surgical orthodontic treatment comprised pre-surgical orthodontic treatment, bilateral sagittal split ramus osteotomy and post-surgical orthodontic treatment. Inclusion criteria were the following: Class III malocclusion was defined according to a value of − 4 mm or more reverse overjet, and all the patients had not received any orthodontic treatments before this study. Exclusion criteria were the presence of craniofacial syndromes, cleft lip and/or palate, or a history of mental and/or physical disorders. Patients who have been scheduled to receive any of orthodontic treatments unrelated to our research were also excluded. The control group mainly consisted of the patients’ relatives, classmates, or colleagues, and a total of 24 healthy individuals were recruited. All the participants enrolled were in good general health and aged between 18 and 33 years. The Ethics Board of Guangxi Medical University approved this study, and all the subjects provided written consent to participate.

### Data collection and instruments

The OHIP-14 has been translated into Chinese and validated for a Chinese population previously [23]. The OHIP-14 consists of 14 items that evaluate the impact of oral health condition on quality of life. Responses to each item are scaled from 0 to 4, which represents “never”, “hardly ever”, “occasionally”, “fairly often” and “very often”, respectively. The total OHIP-14 scores range from 0 to 56 and are obtained by summing the grades for the 14 items. The higher the scores, the lower the oral health related quality of life. The OQLQ was not available in Chinese and it was translated into a Chinese version. This questionnaire consists of 22 items that are graded from 0 to 4, representing “does not bother you at all” to “bothers you a lot” and assess the impact of one’s dentofacial deformity on quality of life across 4 domains, including facial aesthetics (items 1, 7, 10, 11 and 14, range 0 to 20), oral function (items 2 to 6, range 0 to 20), awareness of dentofacial aesthetics (items 8, 9, 12 and 13, range 0 to 16) and social aspects of dentofacial deformity (items 15 to 22, range 0 to 32). Higher scores on the OQLQ are indicative of poorer quality of life.

The QHIP-14 and OQLQ tools were distributed to all patients, and asked them to complete it under the guidance of the experienced researchers at hospital at 3 times, pre-treatment (*T*_0_), pre-surgical orthodontic treatment (6 to 8 months, *T*_1_) and post-surgical orthodontic treatment (6 to 8 months after surgery, *T*_2_). Furthermore, the OHIP-14 questionnaires were collected from controls to determine the differences in quality of life between patients and controls. Both the time for pre-surgical orthodontic treatment and post-surgical orthodontic treatment were about 6 to 8 months, so the duration of each patients was about 12 to 16 months. Some socioeconomic indicators were obtained, including age, gender, marital status and occupation. In order to reduce bias, the same researcher administered all of questionnaires.

### Statistical analysis

Categorical data were presented as percentage (%) and assessed using chi-square test. Numerical data were described as mean ± standard deviation (SD), and the comparisons in these variables between patients and controls were performed using Mann-Whitney U test. The scores of the two questionnaires between *T*_0_, *T*_1_, *T*_2_ and controls (*T*c) were compared using generalized estimated equation. All the statistical analyses were performed using SPSS software version 22.0 (SPSS Inc. Chicago, IL, USA). A level of *P* < 0.05 was considered as statistical significance.

## Results

A total of 21 patients and 24 controls were recruited at baseline (*T*_0_), and no withdrawals occurred. The patients completed surgical orthodontic treatment successfully, and all questionnaires were collected from the participants. The demographic characteristics of the participants are shown in Table 1. The mean age of patients and controls were 24.10 years (SD, 3.67) and 24.42 years (SD, 5.31), respectively. The patient group enrolled 21 subjects, of which 11 were male, accounting for 52.4% of the patients, and the control group comprised 24 individuals, of which 9 were male, representing 37.5% of the healthy controls. The differences in age, gender, marital status and occupation were not statistically significant between the two groups (*P* > 0.05).

**Table 1:** Demographic characteristics of the participants

Variables	Patients *n =* 21	Controls *n =* 24	*P*-*value*
Gender (%)			0.316
Male	11(52.4)	9(37.5)	
Female	10(47.6)	15(62.5)	
Age (years)	24.10 ± 3.67	24.42 ± 5.31	0.854
Marital status (%)			0.905
Unmarried	17(81.0)	18(75.0)	
Married	4(19.0)	6(25.0)	
Occupation (%)			0.847
Student	11(45.8)	11(52.4)	
Office clerk	10(41.7)	7(33.3)	
Self-employed worker	3(12.5)	3(14.3)	

**Fig. 1:**
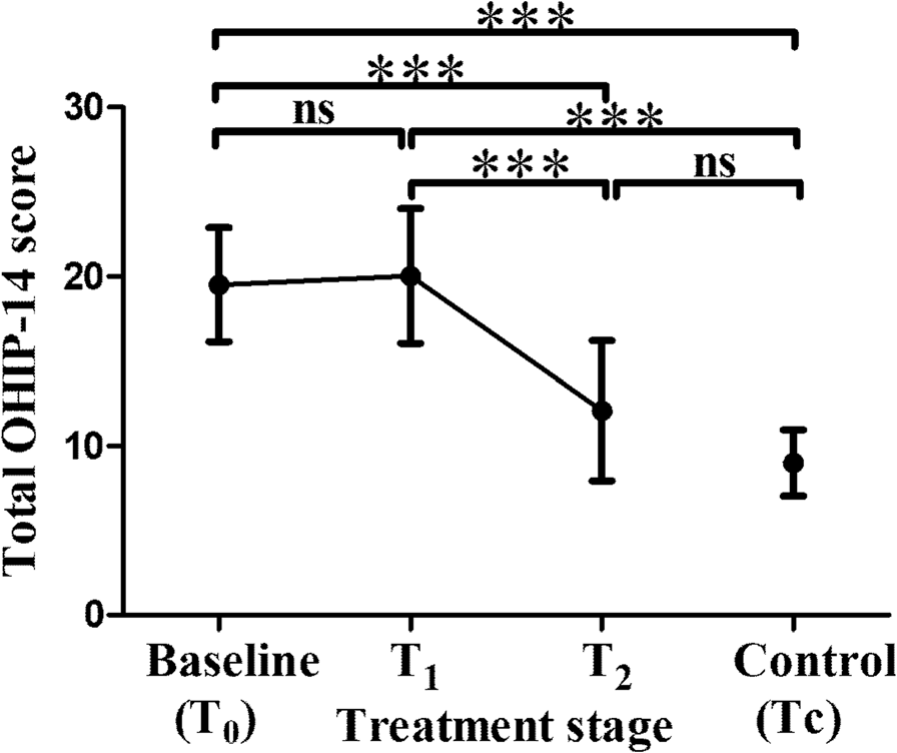
Comparisons in the values of OHIP-14 between *T*_0_, *T*_1_, *T*_2_ and *T*_2_. General estimating equation was used for the comparison of the difference between *T*_0_, *T*_1_, *T*_2_ and *T*_2_ to adjust for age, gender, marital status and occupation. Values indicate mean OHIP-14 score and error bar indicates standard deviation. ns no significance *** *P* < 0.001

Figure 1 shows the changes in the values of OHIP-14. The mean scores in *T*_0_ and *T*_1_ were higher than those in *T*_2_ and *T*c (*P* < 0.001), and a significant decrease was observed after post-surgical orthodontic treatment, which achieved a level similar to the control group (*P >* 0.05).

**Fig. 2:**
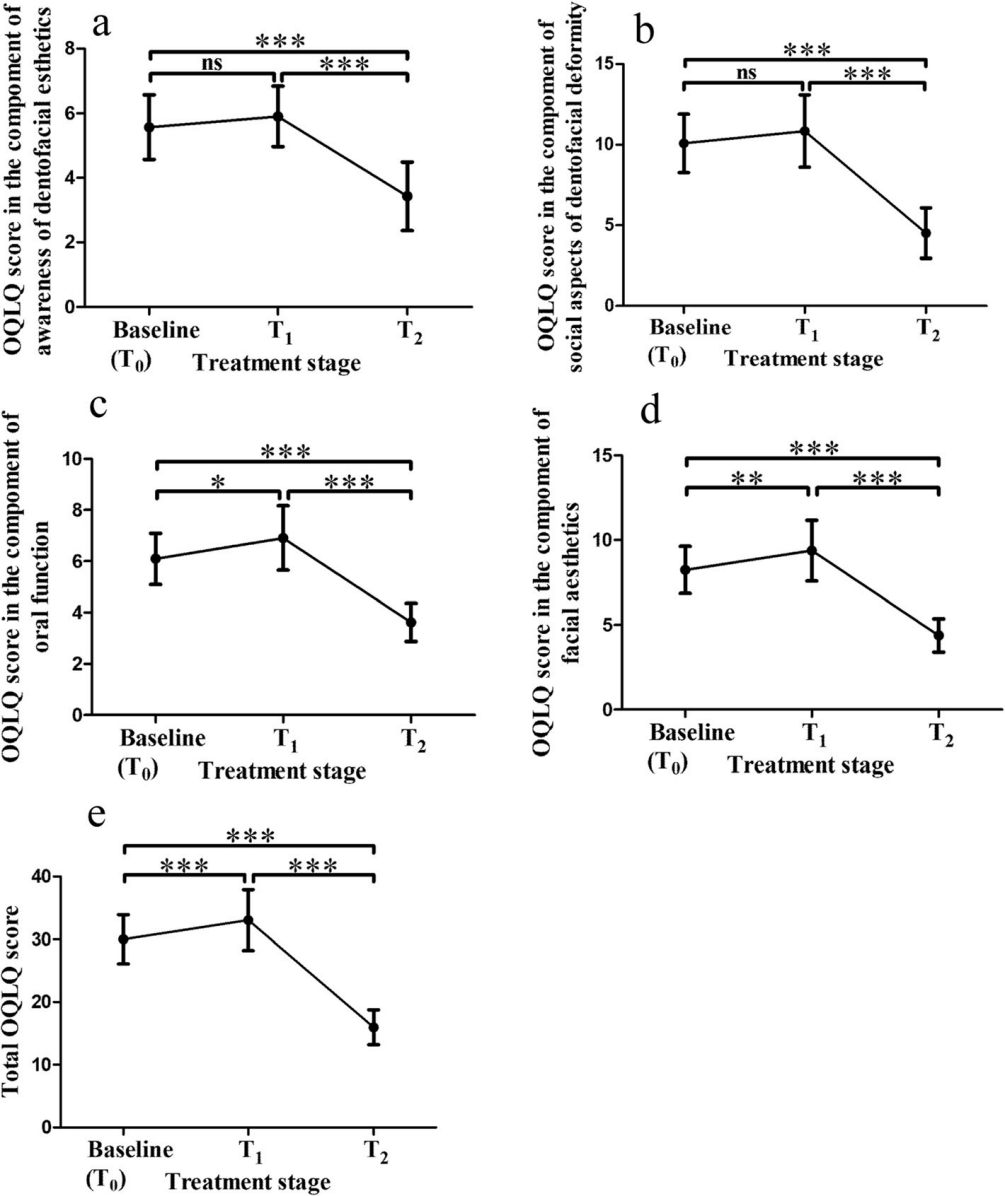
Comparisons in the values of OQLQ between *T*_0_, *T*_1_ and *T*_2_. General estimating equation was used for the comparison of the difference between *T*_0_, *T*_1_ and *T*_2_ to adjust for age, gender, marital status and occupation_._ Values indicate mean OQLQ score and error bar indicates standard deviation. ns no significance * *P* < 0.05 ** *P* < 0.01 *** *P* < 0.001. **a** Comparison of OQLQ score in the component of awareness of dentofacial ethetics during treatment. **b** Comparison of OQLQ score in the component of social aspects of dentofacial deformity during treatment. **c** Comparison of OQLQ score in the component of oral function during treatment. **d** Comparison of OQLQ score in the component of facial aesthetics during treatment. **e** Comparison of total OQLQ score during treatment

The changes in the scores of OQLQ are presented in Fig. 2. The mean scores of all domains showed a significant increase between *T*_0_ and *T*_1_ except for awareness of dentofacial aesthetics (Fig. 2a) and social aspects of dentofacial deformity (Fig. 2b), followed by a decrease between *T*_1_ and *T*_2_.

## Discussion

The present study investigated the changes in quality of life in patients with Class III malocclusion after surgical orthodontic treatment using generic oral health and condition-specific approaches. According to the results of the OHIP-14 questionnaire, the quality of life in patients with Class III malocclusion significantly improved after post-surgical orthodontic treatment, but pre-surgical orthodontic treatment had no effect on quality of life. As to the OQLQ, pre-surgical orthodontic treatment exerted a negative impact on quality of life, especially on the domains of oral function and facial aesthetics, and all the domains significantly improved after post-surgical orthodontic treatment.

To date, surgical orthodontic treatment has been widely applied for the treatment in patients with Class III malocclusion [9, 24]. As the concept of quality of life appears, some studies have been conducted to investigate the effect of surgical orthodontic treatment on quality of life, and there is considerable evidence that surgical orthodontic treatment would bring about great improvements in quality of life [10, 19, 25–29]. In our study, we used OHIP-14 and OQLQ to examine the effects of surgical orthodontic treatment on quality of life in patients with Class III malocclusion, and found that quality of life in patients improved significantly after treatment and was similar to those in controls in OHIP-14, indicating that patients with Class III malocclusion can benefit a lot from surgical orthodontic treatment. Previous studies have reported the significant improvement in quality of life in patients with Class III malocclusion measured by OHIP-14 after surgical orthodontic treatment [30–32]. Baherimoghaddam et al. [19] suggested a continuing improvement process in Class III malocclusion, but a moderate worsening in Class II malocclusion during surgical orthodontic treatment. Silva et al. [33] also observed an increase in the scores of OHIP-14 during treatment. Sun et al. [29] investigated the impact of orthognathic surgery on quality of life in Chinese patients with Class III malocclusion, and observed a significant improvement after treatment, which was significantly better than the control group. The reasons for these inconsistent results may be caused by the different statistical analyses, confounding factors and cultural differences. To help control bias, the current study used generalized estimating equation for the analysis of association of the treatment with quality of life as it allowed for adjustment of participants’ age, gender and other socioeconomic characteristics, and this process may investigate the effect of treatment more properly.

In terms of OQLQ questionnaire, the scores of all domains increased at *T*_1_, this difference was statistically significant for facial aesthetics (*P* < 0.01) and oral function (*P* < 0.05), but decreased significantly across all domains after post-surgical orthodontic treatment. Previous studies have also reported this unexpected observation [22, 34, 35], suggesting that quality of life in patients with Class III malocclusion may be worsened during the treatment. The cause of this phenomenon may be due to the psychosocial disadvantages [36] and the worsening of malocclusion [37–39] during pre-surgical orthodontic treatment. It is reported by most patients that the dental decompensation has an adverse effect on facial aesthetics and this is one of the most stressful periods during the overall treatment [39]. Tachiki and colleagues [35] suggested from their results that there is a transient worsening of quality of life at the post-orthodontic stage, and underlined that dental professionals need to be aware of this and should be used to inform patients about what to expect and provide support to help patients overcome this temporary negative effect of treatment.

In the present study, we observed a significant improvement in quality of life in all domains in OQLQ at post-surgery stage, and these results are in accordance with earlier studies [40, 41]. However, Lee et al. reported the improvements of all domains except for awareness of dentofacial aesthetics at postoperative 6 months [26]. Choi et al. [10] and Tachiki et al. [35] also observed the same findings at the same stage. Cultural and social differences are one of the possible reasons for these results. Most participants in our study were young adults with the pressures of job seeking, establishing friendships, and more personal relationships, and surgical orthodontic treatment may have positive influence on these to some extent. Our patient sample seemed to have a greater representation of males than is commonly reported for studies conducted in Western populations. However, there has been a fervent debate about the prevalence of malocclusion between genders. Some studies suggested a significant higher risk of malocclusion in females [2], but others have reported the opposite observation [42]. Some studies conducted in China have reported no association between gender and prevalence of malocclusion [43, 44]. A systematic review on malocclusion in mainland China from 1988 to 2017 demonstrated that gender may have no significant impact on malocclusion [45]. Additionally, Lin et al. [38] carried out a study on quality of life in Chinese undergraduates, and observed that there is a significant gender difference in OHIP-14 scores only in individuals with mild symptoms, and the OHIP-14 scores would not change between men and women in subjects with moderate or severe symptoms. This result may demonstrate that the negative effect of severe malocclusion on genders may not differ and men have the same willingness to seek treatment. In summary, gender may not be associated with the prevalence of malocclusion and the willingness to seek treatment in patients with Class III malocclusion.

The OHIP-14 and OQLQ have been used in many studies to evaluate the quality of life in patients with dentofacial deformities [14, 17, 29]. Lee et al. [14] used these two tools to investigate the variation of quality of life among patients with dentofacial deformities and controls, and Tajima et al. [46] also investigated the differences in quality of life between patients in need of surgical treatment and those who did not need it. Another study also compared the usefulness of the Short-Form Health Survey (SF-36) and OQLQ in a Jordanian population [47]. Although SF-36, OHIP-14 and OQLQ discriminated different groups well, the OQLQ proved to be more sensitive than the OHIP-14. Previously, OHIP-14 and SF-36 were identified to be insensitive in specific clinical conditions, for instance, in patients with dentofacial deformities [14] or those before and after surgical intervention [22]. In our study, limited change was observed between pre-treatment and pre-surgical orthodontic treatment with the OHIP-14, but when the OQLQ was used, oral function and facial aesthetics exhibited a significant worsening after pre-surgical orthodontic treatment. These different findings between the measurement tools indicates better sensitivity of the OQLQ in the measurement of quality of life in the patients treated. In conclusion, despite the transient worsening of quality of life during pre-surgical orthodontic treatment, surgical orthodontic treatment ultimately appears to promote patients’ quality of life considerably.

Limitations of this study were the small sample size and an insufficient follow-up time, since previous study has pointed towards the long-term effects of orthognathic surgery on quality of life. Finally, the OQLQ questionnaire used in the present study was translated into a Chinese version and this may have some impacts on the results. Further studies on the validation of the Chinese version of OQLQ should be conducted, and more participants should be recruited to confirm these observations.

## Conclusions

Our study showed pre-surgical orthodontics can have temporary negative impact on quality of life in a sample of Chinese young adults with Class III malocclusion. However, after the surgical orthodontic phase of treatment quality of life greatly improved for these patients and their oral health was comparable to control subjects.

## Abbreviations

OHIP-14: The Oral Health Impact Profile
OQLQ: The Orthognathic Quality of Life Questionnaire
SD: Standard deviation
SF-36: Short-Form Health Survey
